# Respiratory syncytial virus disease morbidity in Australian infants aged 0 to 6 months: a systematic review with narrative synthesis

**DOI:** 10.1186/s12889-023-17474-x

**Published:** 2023-12-21

**Authors:** Alice Self, Joseph Van Buskirk, Jayden Clark, Johanne Elizabeth Cochrane, Luke Knibbs, John Cass-Verco, Leena Gupta

**Affiliations:** 1 Sydney Local Health District, Sydney, NSW Australia; 2 School of Public Health, The University of Sydney, Sydney, NSW Australia; 3 Public Health Research Analytics and Methods for Evidence, Public Health Unit, Sydney Local Health District, Sydney, NSW Australia; 4 Department of Paediatrics, Royal Prince Alfred Hospital, Sydney, NSW Australia

**Keywords:** Respiratory syncytial virus, Infants, Morbidity, Mortality, Hospital, Intensive care, Oxygen, Respiration

## Abstract

**Background:**

A significant proportion of the global respiratory syncytial virus (RSV) associated morbidity is accounted for by infants aged 0 to 6 months, who are particularly vulnerable to severe disease. In 2015, 44% of global hospitalisations in infants in this age group were secondary to RSV. The objective of this systematic review is to appraise and synthesise the local evidence of RSV infection morbidity among Australian infants aged 0 to 6 months and to assess the implications for future immunisation strategies.

**Methods:**

Electronic databases (Medline, Embase, Pubmed and Global Health) were searched for full-text articles published between 2000 and 2023 in English language. Studies that examined markers of RSV disease morbidity in infants aged 0 to 6 months in Australia who had laboratory confirmed RSV infection were eligible for inclusion. The outcomes of interest were incidence, prevalence, testing rate, positivity rate, mortality, emergency department visits, community health visits, hospitalisation, intensive care unit admission, supplementary oxygen use, mechanical ventilation, risk factors for disease severity and monoclonal antibody use.

**Results:**

The database search identified 469 studies. After removal of duplicates and full-text review, 17 articles were eligible for inclusion. This review was reported according to the Preferred Reporting Items for Systematic Reviews and Meta-Analyses and Synthesis without meta-analysis guidelines.

**Conclusions:**

Qualitative analysis of the included studies showed that Australian infants aged 0 to 6 months have higher rates of RSV testing, positivity and incidence; and more likely to develop severe disease that requires hospitalisation, intensive care unit admission or respiratory support, compared to children and adults of all ages. Aboriginal and Torres Strait Islander infants aged 0 to 6 months demonstrated higher rates of RSV infection and hospitalisation, compared to non-Indigenous infants. Age-related trends persisted in geographic areas with varying seasonal transmission of RSV, and during the SARS-CoV-2 pandemic. Passive immunisation strategies targeting infants in their first 6 months of life, either via vaccination of pregnant women or administration of long-acting monoclonal antibody during infancy, could effectively reduce RSV disease burden in Australia.

**Supplementary Information:**

The online version contains supplementary material available at 10.1186/s12889-023-17474-x.

## Background

Respiratory Syncytial Viral (RSV) infection is the most common cause of viral acute lower respiratory tract infections globally in children 5 years and younger and is a leading contributor to global morbidity and mortality burden in this age group [1, 2]. In 2019, RSV infections were responsible for 33 million episodes of pneumonia globally and 3.6 million hospital admissions between the ages of 0 to 5 years, of which 39% occurred in infants aged 0 to 6 months [2]. In infants aged 0 to 6 months, 1 in every 28 deaths from any cause globally were secondary to RSV infection [2].

Infants aged 0 to 6 months are at higher risk of severe RSV disease. Infants in this age group have the highest rates of RSV hospitalisation [2, 3] and are more likely to need oxygen supplementation and respiratory support during an acute RSV infection [4]. Furthermore, age 0 to 6 months is an independent risk factor for severe disease in otherwise healthy infants [5] and infants in this age group have the highest mortality rate secondary to RSV infection [6]. In 2019, 51% of all in-hospital deaths secondary to RSV globally occurred in infants aged 0 to 6 months [2].

RSV immunisation programs have the potential to substantially reduce the disease burden in infants aged 0 to 6 months. At present in Australia the only option for RSV prevention is via passive immunoprophylaxis with a monoclonal antibody injection called palivizumab. This is approved only for children at high risk of severe disease, including ex-premature infants, those with chronic lung disease, congenital heart disease or severe immunocompromise [7]. Protection is dependent on maintaining adequate serum concentrations of palivizumab throughout the RSV season; a total of 3 to 6 doses are required at monthly intervals throughout the season and this schedule is repeated annually [8]. This is associated with a significant financial and human cost and has been a barrier to widespread use [9].

RSV vaccines have been in development since the 1960s and offer an opportunity for community-wide protection against disease. In May and July 2023, the United States (US) Food and Drug Administration approved a number of newly developed vaccines against RSV based on the findings of recently completed Phase III randomised controlled trials (RCT). An RCT conducted in 18 countries administered an RSV prefusion F protein-based vaccine (RSVpreF) to over 3500 women in their second half of pregnancy. The trial reported that the vaccine was 81.8% effective in protecting infants from acute RSV infection in first 90 days of life and 69.4% effective in the first 6 months of life [10]. A Phase III trial of an alternative RSV vaccine found a 44.4% reduction in RSV-related hospitalisation in infants of vaccinated mothers in their first 90 days of life [11]. These trials have indicated that passive immunisation via maternal vaccination during pregnancy is effective in protecting infants against severe infection requiring hospitalisation [10, 11]. Based on these findings, an RSV vaccine has now been approved for use in pregnant women in the US and is currently under priority review for approval for use in children [12]. An additional RSV vaccine has been approved for use in adults aged 60 years and over in the US [13].

Another method of passive immunisation that targets infants aged 0 to 6 months is via administration of newly developed monoclonal antibody, Nirsevimab. This is the first single-dose long-acting monoclonal antibody for RSV, demonstrated to be 80.6% effective in preventing RSV requiring hospitalisation when administered to infants aged under 8 months of age prior to their first winter season [14]. Nirsevimab has been approved in Europe, United Kingdom, US and Canada for use in all infants during their first RSV season [15]. Currently, no RSV vaccine or long-acting monoclonal antibody has been approved for use in Australia.

In preparation for future local RSV immunisation programs, an evidence-based allocation scheme must be formulated that prioritises population subgroups who will benefit most. Globally, infants aged 0 to 6 months are considered a priority group for RSV prevention. There is currently no systematic summary of the local evidence on RSV disease morbidity in Australian children in this age group. The importance of examining local data is to determine whether global age-related patterns of RSV infections and outcomes are replicated in Australia. Geographical differences in meteorological factors may affect the seasonality of RSV transmission in Australia, both via altered conditions for viral growth and changes in weather-related human behaviour and living habits [16]. Furthermore, seasonal birth patterns, housing conditions, levels of maternal smoking during pregnancy, number of preterm or low birth weight neonates, maternal parity, socioeconomic status and nutritional status have all been shown to influence RSV outcomes [17–19]. These demographic differences may result in a unique RSV risk profile in Australian infants, distinct from that seen in other countries. Lastly, a summary of local evidence is crucial so that the scope, strengths and gaps of the evidence are mapped in order to provide a basis for informing future research, policy and practice. This will also have implications and relevance for other high-income countries with similar demographic risk profiles and could inform decisions in these settings.

The aims of this systematic review are firstly to identify, synthesise and evaluate studies of RSV morbidity and mortality in Australian infants aged 0 to 6 months, secondly to critically review the strength and scope of the current evidence base, identify gaps in the literature and highlight areas that require further research and lastly to contribute to an evidence base supporting the need for a routine RSV immunisation program targeting infants in their first 6 months of life.

## Methods

### Search strategy

This systematic review was reported according to the Preferred Reporting Items for Systematic Reviews and Meta-Analyses (PRISMA) guidelines (see Additional File 5 for checklist). The search was conducted in four databases, Medline (Ovid), Embase (Ovid), Global Health (Ovid) and Pubmed. The search was limited to studies published between January 1, 2000 and April 12, 2023 that reported on RSV infection morbidity and mortality in Australian infants aged 0 to 6 months. Studies were limited to those published in English. Age-specific search terms were excluded from the search strategy so as to capture studies of all age groups that may have included sub-group analyses within the 0–6 month age range (see Additional file 1).

The outcomes considered for inclusion in this review were: incidence, prevalence, testing rates, positivity rates, mortality, hospitalisation, emergency department (ED) visit, community health visit, intensive care unit (ICU) admission, supplementary oxygen use, mechanical ventilation, risk factors for disease severity and monoclonal antibody (palivizumab) use.

### Selection criteria

#### Participants

Inclusion criteria:

1. Study population includes infants aged 0 to 6 months, or any age sub-groups between 0 and 6 months.
2. Data / findings must be able to be attributable to the 0 to 6 month age group.
3. Infants residing in Australia at the time of RSV diagnosis.
4. Infants who have laboratory-confirmed RSV infection (any diagnostic test) with or without accompanying clinical symptoms of an acute lower respiratory tract infection.
5. Both RSV-A and RSV-B and RSV unspecified confirmed on laboratory diagnosis.

Exclusion criteria:

1. Infants without laboratory confirmed RSV (clinical infection only).

#### Study characteristics

Inclusion criteria:

1. Observational and experimental studies, including cross-sectional studies, case-control studies, clinical trials, cohort studies, case studies, randomised controlled trials, health surveys, ecological studies, as well as narrative reviews, books, book chapters and modelling studies.
2. Both prospective and retrospective studies.
3. Full-text articles.
4. Studies examining RSV-associated acute lower respiratory tract infection short and long-term morbidity and mortality in infants 0 to 6 months of age (or any age subsets within 0 to 6 months).
5. Data gathered between 1 January 2000 and 12 April 2023.
6. Data gathered in Australia only.
7. Studies published in English language only.

Exclusion criteria:

1. Case reports, in vitro studies, animal studies, letter to the editor, correspondence, qualitative thematic analysis, conference proceedings, symposium proceedings, panel discussion, expert opinions, abstract-only, online articles, newspaper articles, oral presentations, guidelines.
2. Studies with less than 10 laboratory-confirmed cases of RSV.
3. Literature reviews, systematic reviews and meta-analyses that report data that was reported previously by another study.

### Literature selection and data extraction

**Fig. 1:**
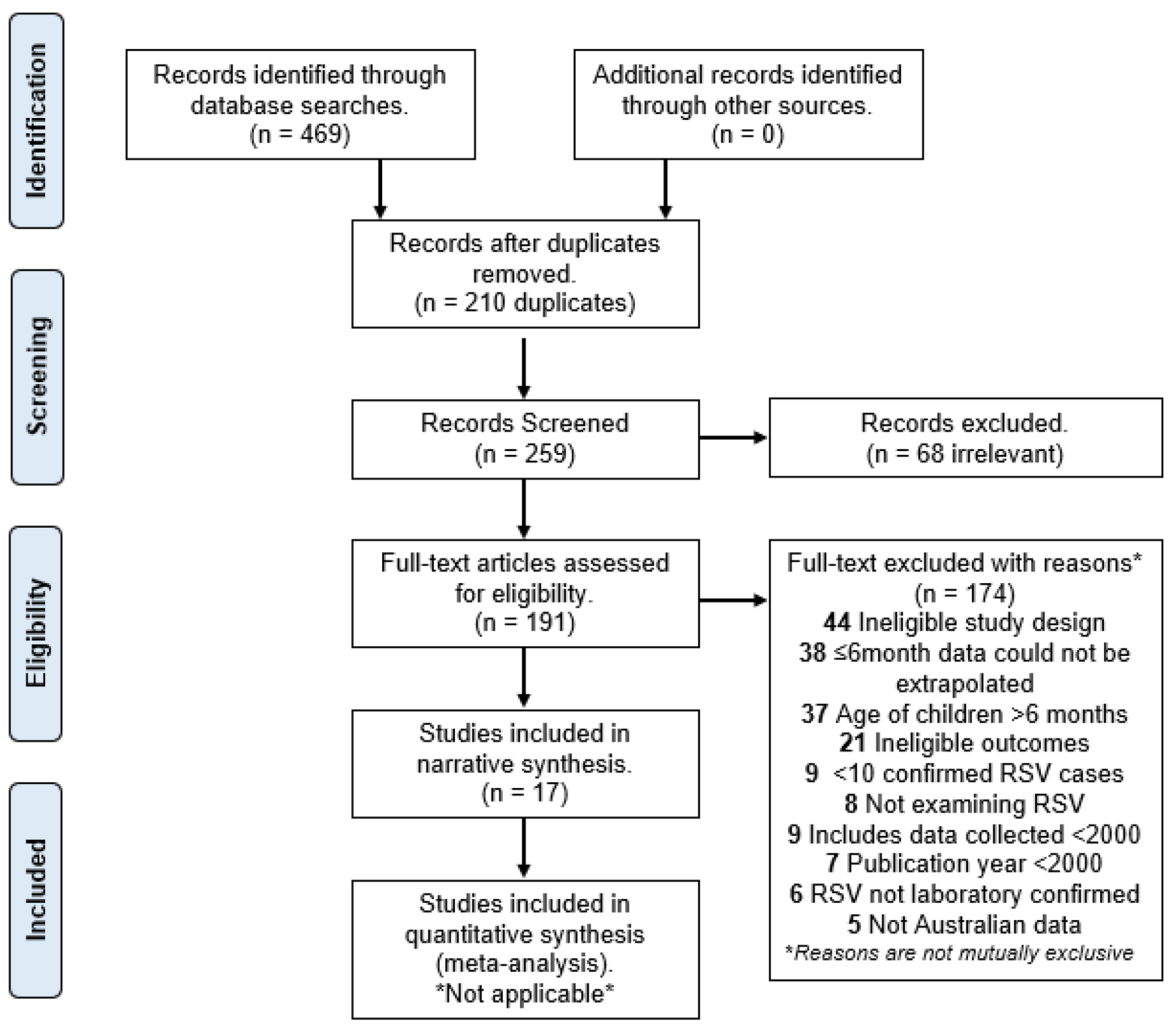
Flow chart summary of the methodology RSV: Respiratory syncytial virus

After removal of duplicates, the titles and abstracts were screened for relevancy. Following this, two independent reviewers (AS, JC) screened abstracts and full texts for inclusion in the review. Any inconsistencies were resolved with a third independent reviewer, JEC. A standardised questionnaire was developed to document the screening and eligibility assessment and record reasons for inclusion or exclusion of studies (Additional file 2). The outcome of this process is summarised in Fig. 1. A large number of studies included infants aged 0 to 6 months as part of a wider population, however, did not report age-specific data for infants aged 0 to 6 months.

After studies were selected for inclusion, a second questionnaire was developed to extract information regarding general study characteristics, population, sample, measured outcomes and results (see Additional file 3). Following this, a narrative synthesis of results was conducted. This information is presented in Table 1. The data extracted from included studies was not amenable to meta-analysis due to significant heterogeneity in measured outcomes. A synthesis without meta-analysis (SWiM) was reported in accordance with the SWiM guideline as an extension to PRISMA guidelines [20]. Results were presented according to the measured outcome of interest related to RSV disease burden.

**Table 1:** Summary of studies of laboratory-confirmed RSV morbidity in infants aged 0–6 months

Study	Region	Objectives	Study design	Population	Sample size	Outcome measures	Findings
Moore et al. (2020) [23]	WA	Describe age-specific epidemiology of RSV infections, including clinical severity and outcomes	Retrospective cohort study	Community, ED, general hospital ward, ICU 2000–2012	469,589 (birth cohort) 45,699 children with RSV testing record 0–16 years	Age-specific RSV testing and positivity rates (per 1000 child-years)	- Overall positivity rate highest in age < 3 months (31, 95% CI 30.2–32.5) - Hospitalised positivity rate highest in age < 3 months (28, 95% CI 27.1–29.2) - Community positivity rates highest in age 3–5 months (1.6, 95%CI 1.4–1.9) - ED positivity rates highest in age 3–5 months (2.3, 95%CI 2-2.7). - Overall testing rate highest in < 3 months (141, 95%CI 138.7-143.6) and second highest in 3–5 month (78.1, 95%CI 76.3–80) - Majority of hospitalisations in age < 6 months (48.2% Indigenous and 48.1% non-Indigenous)
Homaira et al. (2019) [29]	NSW	Investigate an association between hospitalised RSV between 0–2 years old and risk of subsequent asthma	Retrospective cohort study	General hospital ward, ICU 2001–2011	888,154 children (birth cohort) 18,402 infants with RSV hospitalisation 0–2 years	Age distribution of RSV hospitalisations; Age-specific asthma hospitalisation incidence rate (per 1000 child-years)	- 34% of RSV hospitalisations in age < 3 months, 57% in age < 6 months - Incidence rate of asthma following RSV for age < 3 months was 0.5 (95% CI 0.2–0.7), age 3–6 months was 0.9 (95% CI 0.5–1.3) - Rate ratio for asthma hospitalisation was 2–7 fold greater in age > 6 months, compared to < 6 months
Gebremedhin et al. (2022) [22]	WA	Describe rate of RSV positivity in hospitalised children, develop model to estimate the true incidence of RSV hospitalisations	Retrospective cohort study	General hospital ward, ICU 2000–2012	321,825 children (birth cohort) 37,784 children with RSV testing record in hospital 0–5 years	Age distribution of RSV testing, percent positivity, and hospitalisation; Age-specific RSV hospitalisation incidence rate and predicted rate (per 1000 child-years)	- Of those tested for RSV, 22.4% were aged < 3 months and 35.8% were age < 6 months - RSV percent positivity was highest in infants aged < 3 months and 3-<6 months (31% and 32% respectively) - Of those hospitalised with RSV, 30% were age < 3 months, 48.9% were < 6 months - Hospitalisation incidence rate highest in age 28 days – 3 months (31.7, 95% CI 30.3–33.1), second highest in age < 28 days (21.3, 95% CI 19.7–23.0) - Predicted hospitalisation incidence rates highest in age < 3 months (43.7, 95% CI 42.1–45.4)
Moore et al. (2019) [25]	WA	Estimate effectiveness of palivizumab for preventing RSV infections in infants admitted to the NICU at birth	Retrospective cohort study, case series analysis	Community, general hospital ward, ICU 2002–2013	24,329 ex-NICU infants 1506 RSV detected 0–2 years	Age distribution of RSV infections	- Majority (52.1%) of RSV infections were in infants aged < 6 months
Anderson et al. (2021) [37]	VIC	Describe clinical, laboratory, demographic characteristics of children hospitalised with RSV to identify factors associated with severity	Retrospective cohort study	General hospital ward, ICU 2017–2019	970 infants hospitalised with RSV 0–2 years	Predictors of severity (level of respiratory support required)	- Age < 2 months was an independent predictor of severe RSV disease (OR 2.3, 95% CI 1.6–3.3), significantly more likely to require HFNP, CPAP, BiPAP or mechanical ventilation
Homaira et al. (2016) [31]	NSW	Provide population-based age-specific rates of severe RSV illness in children, comparing Indigenous status and those with and without risk factors	Retrospective cohort study	General hospital ward, ICU 2001–2010	870,314 children (birth cohort) 16,119 RSV hospitalisations 0–5 years	Age distribution of RSV hospitalisations; Age-specific RSV hospitalisation incidence rate (per 1000 child-years) in children with and without risk factors (BPD, pre-term, LBW)	- 35.5% of hospitalisations in age 0–3 months, 24.0% in 4–6 months, and 59.5% in age 0–6 months - In children without risk factors, hospitalisation incidence highest in age 0–3 months (25.6, 95% CI 23.9–25.3)*, then 4–6 months (16.7, 95% CI 16.2–17.4) - In children with risk factors, hospitalisation incidence highest in infants with BPD aged 4–6 months (275, 95% CI 194.8–378.0) - In indigenous children, hospitalisation incidence highest in ages 0–3 months (58, 95% CI 49.5–60.5)
Lim et al. (2017) [32]	WA	Describe the pathogen-specific burden and age-specific rates of respiratory viruses in a cohort of hospitalised children	Retrospective cohort study	General hospital ward, ICU 2000–2012	469,589 children (birth cohort) 30,859 children with RSV testing record 8769 RSV positive 0–16 years	Age-specific hospitalisation incidence rate (per 100,000 child-years)	- RSV hospitalisation incidence rate highest in age 1–5 months (~ 2400) and < 1 month (~ 2100)
Dede et al. (2010) [34]	NT	Describe seasonality of RSV infection in a desert region, compare age-specific RSV hospitalisation rate	Retrospective case series	General hospital ward 2000–2004	173 infants with RSV hospitalisation < 2 years	Age distribution of RSV hospitalisations	- 11.6% of RSV hospitalisations were in infants aged < 1 month, 29.5% in age < 3 months, 52.6% in < 6 months
Saravanos et al. (2019) [3]	AUS	Estimate the age-specific rates of RSV-associated hospitalisation to identify groups at risk of serious RSV disease	Retrospective cohort study	General hospital ward, ICU 2006–2015	86,687 RSV hospitalisations All ages	Age-specific RSV hospitalisation rates (per 100,000 population) compare Indigenous and non-Indigenous; Hospitalisation risk	- Highest hospitalisation incidence rate in infants aged 0–2 months (3129), second highest in age < 6 months (2468) - RSV hospitalisation rate higher in Indigenous versus non-Indigenous infants aged < 6 months (4310 versus 2253) - Risk of RSV hospitalisation was 1.9 times higher in Indigenous infants compared to non-Indigenous infants age < 6 months (IRR 1.9, 95% CI 1.8-2.0).
Saravanos et al. (2022) [24]	NSW	Characterise the magnitude, severity and age-related changes of the shifted RSV epidemic during the SARS-CoV-2 pandemic compared to pre-pandemic	Ecological study	Community, ED, General hospital ward, ICU 2014–2020	22,997 RSV infections 0–16 years	Age distribution of RSV infections, hospitalisations, ICU admissions (annual average count); RSV test positivity	- Pre-SARS-CoV-2 (2014–2019), RSV infections highest in age 0–5 months (453 cases, SD 43.15) - Hospitalisations highest in age 0–5 months (454, SD 32.25) - ICU admissions highest in age 0–5 months (131, SD 18.76) - Annual counts quadruple that seen in age 6–11 months - Age-related trends persisted in 2020 during SARS-CoV-2 pandemic - Highest RSV test percent positivity was in infants aged 0–5 months (23.82% in 2015–2019, and 19.54% in 2020).
Fagan et al. (2017) [26]	NT	Determine age-specific prevalence of RSV in NT, investigate drivers of seasonality	Retrospective audit	Community, ED, General hospital ward, ICU 2012–2014	272 RSV infections All ages	Age distribution of RSV infections	- 32% of all RSV infections in infants aged < 6 months - 26.8% of infections were in non-Indigenous children aged < 6 months and 36.2% in Indigenous children aged < 6 months - RSV detection is positively correlated with higher rainfall
Butler et al. (2019) [30]	SA, QLD	Determine factors associated with severe RSV disease in hospitalised children	Retrospective cohort study	General hospital ward, ICU 2013–2014	496 RSV infections < 3 years	Age distribution of RSV hospitalisation; Predictors of severity (ICU/ HDU, NG/IV rehydration, LOS > 5 days, oxygen, mechanical ventilation)	- Majority of RSV hospitalisations were in infants aged < 6 months (68.8%) - Age negatively associated with RSV disease severity (OR 0.95; 95% CI 0.90–0.99, *p* = 0.02), decreasing age (months) increases risk of severe disease - Median age for severe hospitalised RSV was 4.7 months (IQR 0.33-6.0)
Pham et al. (2020) [36]	VIC	Describe epidemiology and treatment of RSV infection in a tertiary paediatric ICU	Descriptive case study	ICU 2005–2015	604 RSV infections 0–16 years	Age distribution of ICU admissions	- 43% of RSV infections requiring ICU admission were in infants aged < 3 months, 6% were in infants < 30 days old
Fathima et al. (2018) [33]	WA	Assessed the impact of pneumococcal vaccination on all-cause and pathogen-specific pneumonia hospitalisations	Retrospective cohort study	General hospital ward, ICU 2000–2012	469,589 children (birth cohort) 15,175 pneumonia hospitalisations 1097 RSV positive 0–16 years	Age-specific and pathogen-specific detection rates (per 100,000 child-years)	- In indigenous infants < 6 months, RSV detection rate was highest of all pathogens (488.3) - Highest RSV detection rate was in ages < 6 months (488.3) compared to all other ages. Lower RSV detection rates in non-Indigenous infants < 6 months (80.1)
Nguyen et al. (2023) [35]	WA	To present the number, clinical presentation and severity of RSV-related admissions during a global pandemic.	Ecological study	General hospital ward 2018–2021	294 RSV hospitalisations 0–16 years	Age-distribution of RSV hospitalisation; Severity of RSV infection (respiratory support)	- In 2018, 22.4% of RSV hospitalisations were aged 0–1 month, 2019 there were 16.3% and 2020 there were 5.4% - Significantly lower need for oxygen therapy in 2020 vs. 2018 (*p* = 0.004), due to changed age distribution, lower proportion of 0–1 month age group, most likely to require respiratory support
Moore et al. (2012) [27]	WA	To document the proportion of ALRI hospitalisations with positive identification of a respiratory pathogen.	Descriptive case study	General hospital ward, ICU 2000–2005	19,857 ALRI hospitalisations 8980 with RSV testing record 0–9 years	Pathogen-specific cause of ALRI	- RSV was the most common pathogen identified in infants aged < 6 months admitted with bronchiolitis (63.7%) - RSV was the most common pathogen identified in infants aged < 6 months admitted with ALRI (36.6%)
Chappell et al. (2013) [28]	QLD	To assess the diversity and prevalence of bacterial pathogens associated with viral infections of the respiratory tract in children.	Descriptive case study	General hospital ward, ICU 2012	201 children 0–5 years	Pathogen-specific cause of ALRI	- Overall, RSV was the most common pathogen identified in infants aged 0–6 months with ALRI (34.9% of 89 samples tested). - RSV mono-infection was most common in 0–6 month age group (31.3% of samples), compared to other age groups up to 5 years.

*Note: Error in reporting of results in the original study, with a point estimate that lies outside of the confidence interval

Abbreviations: ALRI, acute lower respiratory tract infection; CI, confidence interval; ED, emergency department; HDU, high dependency unit; ICU, intensive care unit; IV, intravenous; LOS, length of stay; NG, nasogastric; NICU, Neonatal intensive care unit; NSW, New South Wales; NT, Northern Territory; OR, odds ratio; QLD, Queensland; RSV, respiratory syncytial virus; SA, South Australia; SARS-CoV-2, Severe Acute Respiratory Syndrome Coronavirus 2; SD, standard deviation; VIC, Victoria; WA, Western Australia

### Quality of evidence appraisal

Quality of evidence was assessed using the CASP checklist for cohort studies [21]. The questionnaire included 14 items, which were divided into 3 sections; section A for validity of results, section B for reporting of results and section C for applicability and generalisability. Each question could be answered as “Yes” (1 point), “No” (0 points) or “Can’t tell” (0.5 points), except item 7, which was an open-ended question. Item number 8 was modified from “How precise are the results?” to “Are the results precise?”, considering the width of the confidence interval, sample size, sample selection and representativeness. The total possible number of points for each study was 13. The outcome of each question was reported for each study in a table (Additional file 4). An overall “score” was reported which classified the quality of evidence (risk of bias) as either low, moderate or high. A score of 11.5 or above was classified as high quality, score of 9–11 was classified as moderate quality and a score of 8-9.5 was low quality. Any study scoring below 8 was excluded from the review.

## Results

### General characteristics

#### Population

This systematic review included 17 eligible publications which collectively analysed the data from infants aged 0 to 6 months from all states and territories in Australia. Data was gathered between 2000 and 2023. The settings varied across studies; community (4/17), ED (3/17), hospital general ward only (2/17), ICU only (1/17) and combined ward and ICU inpatients (14/17).

#### Diagnostic methods

The method of laboratory detection varied; 13/17 studies used polymerase chain reaction (PCR) testing, 5 used viral culture, 7 used direct immunofluorescence antigen testing, 7 studies used a combination of methods and 3 studies did not specify the method of laboratory confirmation. In the studies that did not specify type of laboratory testing performed, ICD-10-AM diagnostic codes were used that specifically required laboratory confirmation of RSV pathogen. Clinical diagnoses accompanied laboratory confirmation of RSV in the majority of studies (15/17 studies). Most studies utilised ICD-10-AM criteria for clinical diagnosis of RSV infection (10/17), with 5/17 studies using other symptom criteria and 2/17 studies did not specify whether clinical symptoms were present.

#### Outcomes

The following RSV-related outcomes were measured; testing rate (2/17), positivity rate (3/17), proportional age distributions of infections (5/17), hospitalisation (9/17), ICU admission (2/17), respiratory support requirement (3/17) and long-term sequelae (1/17).

#### Quality of evidence

Out of the 17 studies included in the analysis, 12 were classified as high quality of evidence, 4 were moderate and 1 was low quality (see Additional file 4). No studies were excluded based on the outcome of quality appraisal.

### RSV epidemiology

#### Testing

Two studies examined RSV testing rates. The first noted that of 37,784 infants between 0 and 5 years tested for RSV during a hospital admission, 35.8% were aged 0–6 months and 22.4% were aged 0–3 months [22]. A further study noted that from a birth cohort of 469,589 children aged 0–16 years, RSV testing rate was highest in infants aged less than 3 months (141 per 1000 child-years, 95%CI 138.7-143.6) [23].

#### Positivity

Positivity was calculated as the rate or proportion of RSV positive results out of the total number of children who underwent RSV testing. One study noted that from 45,699 children aged 0–16 years tested for RSV across community, ED and hospital settings, RSV positivity was highest in infants aged less than 3 months and second highest in infants aged 3–5 months [23]. When examining positivity rates according to clinical severity, infants aged less than 3 months and 3–5 months consistently demonstrated the highest positivity rates in community, ED and hospitalised settings, when compared to children aged up to 16 years [23]. Another study across community, ED and hospital settings noted highest RSV test percent positivity in infants aged 0–5 months at 23.82% in 2015–2019 (pre-SARS-CoV-2) and 19.54% in 2020 [24]. This finding was replicated in a cohort of 37,784 hospitalised infants between 0 and 5 years; RSV percent positivity was highest in infants aged less than 3 months and 3–6 months (31% and 32% respectively) [22].

#### Proportional age distribution

Three studies examined the proportional age distribution of RSV infections in combined community, ED and hospitalised settings. Of 24,329 ex-neonatal intensive care unit (NICU) infants aged 0–2 years with RSV, 52.1% of infections occurred in infants aged under 6 months [25]. Of 22,997 children aged 0–16 years with RSV, the highest annual average count of infections was in infants aged 0–5 months (453) [24]. This was more than double the next highest annual average RSV count in infants aged 12–23 months. A third paper found that of 272 children and adults with RSV, the highest proportion of infections were in infants aged 0–6 months (32%) [26]. Furthermore, of the infections in the Aboriginal and Torres Strait Islander population, 36.2% were aged 0–6 months; in the non-Indigenous population, 26.8% were aged 0–6 months [26].

Two studies examined proportional age-distribution of RSV infections (as compared to other viral or bacterial pathogens) in hospitalised infants. In a cohort of 2521 infants aged 0–6 months hospitalised with bronchiolitis, RSV was the most common pathogen identified, detected in 63.7% of cases in this age group [27]. Comparatively, RSV was less prevalent in older age groups hospitalised with bronchiolitis; detected in only 45% of infants aged 6–11 months and 53.3% in aged 12–23 months [27]. A second study noted that of 83 children aged 0–6 months presenting to hospital with respiratory symptoms during the winter season, RSV was the most common pathogen identified (from a number of viral and bacterial pathogens tested), detected in 34.9% of samples [28].

### RSV hospital morbidity

#### Combined hospitalisation

Ten studies examined RSV hospitalisation, combining general ward and ICU admission in a single cohort.

Six studies commented on the proportional age distribution of RSV hospitalisation. From 18,402 children aged 0–2 years hospitalised with RSV, 34% were less than 3 months old and 57% were less than 6 months [29]. Another study of 45,699 children aged 0–16 years tested for RSV and stratified by Indigenous status found the majority of RSV hospitalisations occurred in infants aged 0–6 months (48.2% Aboriginal and Torres Strait Islander and 48.1% non-Indigenous) [23]. In a cohort of 8,604 infants aged 0–2 years of age hospitalised with RSV, 30.1% were less than 3 months old and 48.9% were aged less than 6 months [22]. In a cohort of 496 infants aged 0–3 years hospitalised with RSV, 68.8% of these hospitalisations were in infants aged less than 6 months [30]. From a cohort of 16,119 infants hospitalised with RSV, the highest proportion of hospitalisations were in infants aged 0–6 months (59.5%) [31]. In a study of 22,997 children aged 0–16 years hospitalised with RSV, the highest number was in infants aged 0–5 months (annual average count of 454, SD 32.25), which was more than 2.5 times greater than the next highest average annual count of 175 in 6–11 month age group [24].

Five population-based studies calculated age-specific RSV hospitalisation incidence. From a birth cohort of 321,825 children aged 0–5 years, RSV hospitalisation incidence was calculated to be highest in infants aged 1–3 months (31.7/1000 child years, 95% CI 30.3–33.1) and second highest in infants aged less than 28 days (21.3/1000 child-years, 95% CI 19.7–23.0). Comparatively, the incidence rate for 6–12 month olds was markedly lower at 9.5/ 1000 child-years [22]. A second study calculated RSV hospitalisation incidence for infants with and without risk factors for severe disease, from a birth cohort of 870,314 children aged 0–5 years [31]. In healthy children with no risk factors, RSV hospitalisation incidence was highest in infants aged 0–3 months (25.6/ 1000 child-years, 95% CI 23.9–25.3), with incidence negatively associated with age up to 5 years [31] (please note the error in the original study with a point estimate that lies outside of the confidence interval). In infants with risk factors, highest incidence of RSV hospitalisation is in infants with bronchopulmonary dysplasia (BPD) aged 4–6 months (275/1000 child-years, 95% CI 194.8–378.0) [31]. Comparatively, in infants aged 7 months to 5 years, incidence was < 100/1000 child-years. In Aboriginal and Torres Strait Islander children (both high risk and healthy), incidence was again highest in infants aged 0–3 months (58/1000 child-years, 95% CI 49.5–60.5) [31]. This is notably lower than older age groups, with a rate of only 9.8/1000 child-years in infants aged 7–11 months (95% CI 8.7–11.1) [31]. Another study calculated RSV hospitalisation incidence from a birth cohort of 469,589 children aged 0–5 years [32], again noting highest incidence in infants aged 1–5 months (~ 2400/ 100,000 child years) compared to all other age groups [32]. The next study examined a cohort of 86,687 children and adults of all ages with RSV, reporting the highest hospitalisation incidence in infants aged 0–2 months (3129/100,000 population) and second highest in infants aged 0–6 months (2468/100,000 population) [3]. These rates were markedly higher than the next highest, at 500/100,000 population in children aged 12–23 months. They also found that the number of RSV hospitalisations in children less than 5 years of age peaked at 1 month old, then steadily declined thereafter [3]. When comparing Aboriginal and Torres Strait Islander and non-Indigenous infants aged less than 6 months, RSV hospitalisation risk was 1.9 times higher in Aboriginal and Torres Strait Islander compared to non-Indigenous infants in this age group (IRR 1.9, 95% CI 1.8-2.0). The final study calculated RSV-specific pneumonia hospitalisation rates from a birth cohort of 469,589 children aged 0–16 years, stratified by age and Indigenous status [33]. From this sample, there were 15,175 pneumonia hospitalisations. In Aboriginal and Torres Strait Islander infants aged less than 6 months, RSV-specific hospitalisation rate was the highest of all pathogens tested, at 488.3/ 100,000 child-years. This was 1.6 times higher than the next most common pathogen-specific rate of Picornavirus (at 295.6/ 100,000 child-years). Similarly, in non-Indigenous infants aged less than 6 months, RSV-specific hospitalisation rates were the highest (80.1/ 100,000 child years), which was 6 times higher than the next most common pathogen. Overall, RSV detection rates decreased with increasing age [33].

#### General ward

Two studies examined RSV hospitalisation specifically in a general paediatric ward (excluding ICU admissions). A retrospective case audit of 173 infants aged less than 2 years found that 11.6% of admissions were in infants < 1 month old, 29.5% were < 3 months and 52.6% were in infants <.

6 months old [34]. A second retrospective observational study of 294 children between aged 0–16 years hospitalised with RSV in 2018, reported that 22.4% of hospitalisations were infants 0–1 months [35]. Furthermore, they noted that in 2019 and 2020 during the SARS-CoV-2 pandemic, the proportion of RSV hospitalisations in infants aged 0–6 months decreased to 16.3% and 5.4% respectively, with a more even distribution of infections between ages 0–5 years [35].

#### ICU

One study examined the proportional age-distribution of RSV requiring ICU admission. In a cohort of 604 children aged 0–16 years admitted to ICU with RSV, 6% of admissions were in infants aged < 30 days and 43% in infants aged < 3 months [36]. A second study performed a subgroup analysis of infants admitted to the ICU who were part of a larger cohort of 22,997 children aged 0–16 years hospitalised with RSV [24]. The highest annual average count of RSV requiring ICU was in infants aged 0–5 months (131, SD 18.76), more than triple the next highest count of 34 in infants aged 6–11 months [24].

#### Respiratory support

Two studies examined the association between age and respiratory support requirement during an RSV infection. In a cohort of 970 infants aged 0–2 years hospitalised with RSV, age less than 2 months was an independent predictor of severe RSV [37]. The odds of requiring high flow nasal prongs (HFNP), continuous positive airway pressure (CPAP), bilevel positive airway pressure (BiPAP) or mechanical ventilation was 2.3 times higher in infants aged less than 2 months compared to all other ages up to 2 years (OR 2.3, 95% CI 1.6–3.3, *p* < 0.0001) [37]. A second cohort of 496 children aged 0–3 years hospitalised with RSV found that age (in months) was negatively associated with markers of RSV severity [30]. Younger age was associated with increased likelihood of requiring supplemental oxygen, mechanical ventilation, ICU or HDU admission, IV or NG rehydration or hospital length of stay greater than 5 days (OR 0.95; 95% CI 0.90–0.99, *p* = 0.02). The median age for severe hospitalised RSV disease was 4.7 months with an interquartile range (IQR) of 0.33 to 6 months [30].

One study examined proportions of hospitalised RSV requiring respiratory support before and during the SARS-CoV-2 pandemic [35]. From a cohort of 294 children aged 0–16 years hospitalised with RSV, they found a statistically significant reduction in the proportion requiring respiratory support in 2020, compared to pre-pandemic levels in 2018 (*p* = 0.004). This correlated with decreased proportion of RSV hospitalisations occurring in infants aged 0–1 month in 2020 compared to 2018 (5.4% and 22.4% respectively). This age group is typically more likely to require respiratory support during an RSV infection, hence fewer infections in this age group resulted in reduced respiratory support requirement.

#### Seasonality

Three papers examined seasonal variation of RSV infections in infants aged 0–6 months. A study of children aged 0–3 years hospitalised with RSV noted that in the tropical climate of far north QLD, peak RSV infections correlated with higher rainfall during summer, whereas in temperate conditions in SA, peak infections occurred during winter. Despite seasonal variation, the majority of RSV infections were in infants less than 6 months old (68.8%) [30]. Similarly, another study conducted in the tropical region of top-end NT noted peak RSV infections during summer months of higher humidity and rainfall, with highest proportion of infections consistently occurring in infants under 6 months [26]. One further study examined RSV seasonality in an arid, desert climate of central Australia, noting a lower overall incidence and more even distribution of infections across spring to autumn months. Again, the majority of RSV hospitalisations still occurred in infants under 6 months (52.6%) [34].

#### Long-term impacts

One study examined the association between hospitalised RSV between 0 and 2 years old and risk of subsequent asthma [29]. From a birth cohort of 888,154 children aged 0–2 years, 18,402 were hospitalised with RSV. For children who were hospitalised with RSV at 0–3 months of age, the incidence of subsequent asthma was 0.5/1000 child-years and for infants with RSV at 3–6 months of age, asthma incidence was 0.9/1000 child-years. The rate ratio for asthma hospitalisation was 2–7 times greater in children who were 6 months and older at the time of RSV infection, compared to children who had RSV between 0 and 6 months. Thus, RSV at a later age was associated with a higher risk of subsequent asthma hospitalisation [29].

## Discussion

The World Health Organization (WHO) has identified RSV as a priority area for research, specifically regional age-stratified estimates of disease burden, especially in the first months of life [38]. In this systematic review, we appraised published evidence of age-specific RSV disease morbidity, with a focus on infants aged 0 to 6 months. This is the first systematic review to specifically examine the evidence for RSV disease morbidity in Australian infants in this high-risk age group in isolation.

Our review of the evidence indicated that RSV disproportionately affects younger infants, specifically those aged 0 to 6 months, compared to children and adults of all ages. Secondly, infants aged 0 to 6 months are more likely to develop severe RSV disease requiring hospitalisation. Infants aged 0 to 6 months accounted for the majority of RSV hospitalisations and demonstrated consistently higher hospitalisation incidence rates. Thirdly, there is a higher incidence and severity of RSV disease in Aboriginal and Torres Strait Islander infants, compared to non-Indigenous infants aged 0 to 6 months. Next, age-related patterns of RSV persisted during the SARS-CoV-2 pandemic. Lastly, age-related patterns remain the same in regions that demonstrate different seasonality of RSV infections.

### Infants disproportionately affected

This review has verified that infants aged 0 to 6 months are disproportionately affected by RSV across community, ED and hospital settings, compared to children and adults of all ages. Infants in this age group are more likely to be tested for RSV, demonstrate consistently higher RSV test positivity rates and are disproportionately represented when examining the age-distribution of overall RSV infections.

### Greater Disease severity in infancy

Markers of disease severity that were examined included hospitalisation to either a general ward, ICU or HDU, supplemental oxygen use, HFNP, CPAP, BiPAP, mechanical ventilation and length of hospital stay greater than 5 days. Younger age was associated with increased likelihood of severe disease and specifically, age less than 2 months was noted to independently predict severe disease. Only one study distinguished between infants with and without comorbidities and noted a markedly higher incidence of RSV requiring hospitalisation in infants aged 0 to 6 months with comorbid conditions including BPD, prematurity and low birth weight [31]. Again, this pattern may partly reflect a bias towards testing in children who are at risk of severe disease.

Disease severity is likely the result of complex interplay between physiological, anatomical and immune differences. The physiological culmination of the inflammatory response against RSV infection is airway obstruction and formation of mucus plugs [39]. In infants, who have incomplete lung maturation and thus smaller, more narrow airways, this airway obstruction has a greater clinical significance and predisposes them to more severe disease [39]. Disease severity has also been linked to level of maternal antibody to RSV (RSV-mAb) in an infant’s circulation at birth [40]. These neutralising antibodies have a half-life of approximately 2.5 months, after which point the levels decline rapidly and reach a nadir at around 6 months. Passive immunisation by RSV-mAb prior to 6 months is essential in protecting an infant against RSV disease. Level of RSV-mAb has been inversely associated with RSV disease severity [41].Furthermore, children who are infected with RSV in the first 6 months of life have been shown to consistently have lower cord titres of RSV-mAb [40]. Hence, maternal immunisation against RSV during pregnancy is a promising and permissible route to boost circulating RSV-mAb levels during this critical gap of vulnerability in the first 6 months of life [42].

### Higher incidence and severity in Aboriginal and Torres Strait Islander infants

Our review confirmed that rates of severe RSV requiring hospitalisation were higher in Aboriginal and Torres Strait Islander infants aged 0 to 6 months, compared to non-Indigenous infants. These findings reiterate the inequities in health outcomes between Aboriginal and Torres Strait Islander and non-Indigenous children in Australia. This may be attributable to persistent social inequities, including poor access to healthcare services, inadequate housing and higher exposure to tobacco smoke [31]. These findings support the need to prioritise Aboriginal and Torres Strait Islander infants for RSV immunoprophylaxis as part of culturally safe, co-designed and led models of healthcare.

### Impact of the SARS-CoV-2 pandemic

A dramatic decrease in the overall number of RSV infections was observed during the SARS-CoV-2 pandemic, compared to pre-pandemic levels [24, 35]. This was followed by a delayed seasonal RSV transmission pattern after lockdown restrictions were eased, with out of season clustering occurring during the summer months of 2020. A change in age-distribution of RSV infections was noted, with increased numbers in children aged 12 months to 5 years, likely due to the accumulation of RSV-naive older children during the pandemic. Despite this, the 0 to 6 month age group remained the most at-risk, demonstrating the highest counts of RSV infections, hospitalisations and ICU admissions before and during the pandemic [24]. This age group has a physiological vulnerability to infection that persisted despite the environmental and social changes that accompanied the SARS-CoV-2 pandemic. Further studies are required to assess whether, in our current post-pandemic state, the age-distribution of RSV infections has returned to that observed prior, with infections more heavily skewed towards younger infants.

### Impact of seasonal transmission pattern

Age-related patterns also remain consistent in regions of Australia that demonstrate distinct seasonal patterns of RSV transmission. In the NT and far north QLD, RSV transmission is positively correlated with times of higher rainfall and humidity, which tends to occur in the summer (monsoon) seasons. By contrast, in temperate regions of Australia, RSV transmission occurs during winter months. RSV is predominantly transmitted from person to person via direct and indirect contact [43]. Increased rainfall and humidity have been proposed to facilitate RSV transmission via increased deposition of viral particles on surfaced and increasing survival of the virus in droplets on surfaces [43]. Despite altered seasonal transmission patterns, the burden of disease remains highest in infants aged 6 months and younger. Variations in RSV seasonal transmission pattern are important when making decisions about timing of passive immunoprophylaxis, as the months during which infants are at highest risk of infection will vary between climatic regions of Australia.

### Evidence gaps

This review highlighted a number of significant knowledge gaps. Firstly, no studies examined RSV burden in ED settings specifically. A number of studies pooled these presentations with a larger inpatient cohort, however failed to provide information on ED-specific burden. In the US in 2015, upper respiratory infections and disorders were the most common reasons for ED presentation across all paediatric age groups. Infants aged 12 months or younger had the highest rate of ED presentations with a primary diagnosis of upper respiratory tract infection compared to all other paediatric age groups [44]. Furthermore, 96.7% of paediatric ED presentations were treat and release [44]. In Australia between 2017 and 2018, respiratory illnesses were the most common reason for ED presentation in infants aged 0–4 years [45]. A large number of infants presenting to ED with respiratory illnesses, including RSV, would be managed and discharged home, thus consuming a large volume of resources in management even without the need for hospitalisation. There is no current local evidence of the specific burden of RSV management placed on emergency departments in Australia. However, given that respiratory illnesses are the most common reason for ED presentation, reducing RSV disease in infants via passive immunisation program is likely to result in significant reduction in burden placed on ED. Secondly, there is a paucity of data examining RSV burden in infants who are managed in community settings. These gaps are likely related to the lack of historical routine surveillance data.

Furthermore, RSV-related mortality rates in infants aged 0 to 6 months in Australia are poorly understood. Pooled global estimates reported that in 2015 alone there were over 27,000 in-hospital deaths secondary to RSV in infants aged 0 to 6 months [46]. In Australia between 1998 and 2018, the annual in-hospital mortality rate secondary to RSV was 0.6 per 10,000 hospitalised children aged up to 16 years [47]. Between 2006 and 2015, there were only 21 in-hospital deaths secondary to RSV in infants aged 0–5 years [3]. However, these estimates fail to account for out-of-hospital deaths as well as the impact of very young age on mortality risk.

Only one study examined a long-term impact of severe RSV requiring hospitalisation in infants aged 0–6 months [29]. They found that RSV hospitalisation at age 0–6 months was associated with a lower risk of subsequent asthma, compared to children who were older than 6 months at the time of RSV hospitalisation. This was not consistent with prior studies, which noted that infants aged 4 months at the time of non-specific viral bronchiolitis had the highest risk of subsequent childhood asthma [48]. Minimal published data on RSV-specific long-term complications makes comparability of findings difficult. This is an important evidence gap. It is critical that there is a thorough understanding of the association between RSV infection in early life and long-term health outcomes, in order to assess whether immunisation will also protect infants against these long-term term risks.

### Strengths of the study

The strength of this systematic review is that it is first to summarise available evidence of the high burden of RSV disease specifically in Australian infants aged between 0 and 6 months. As previously discussed, RSV prevention to date has relied on monoclonal antibody immunoprophylaxis, only for high-risk infants. This drug is expensive and burdensome to deliver [7, 8]. Furthermore, the healthcare cost associated with lack of routine RSV prevention is significant. A large part of this cost is attributable to high hospitalisation rates in infancy. In Australia, the mean cost per child attributable to a hospital admission secondary to RSV disease is $17,120 per child [49]. The estimated annual national cost of RSV disease in Australia in 2018 was between $59 million and $121 million, which is seven times higher than the cost associated with influenza disease [49]. Hence, preventing severe disease via a routine RSV passive immunisation program targeting infants aged 0–6 months would significantly reduce these healthcare-associated costs.

Further to this, our extensive database search included over a decade worth of locally collected data. All studies included had large sample sizes, thus were sufficiently powered to detect true differences in the population studied. The conclusions drawn from this review in support of the high burden of RSV disease in infancy are based on the majority of studies (12/17) yielding a high grade of quality of evidence. All studies included relied on laboratory confirmation of RSV, which improves the accuracy of estimates. Methods of laboratory detection varied, however, most studies utilised polymerase chain reaction tests. The sensitivity and specificity of viral PCR testing for RSV is high at 98.0% and 94.3% respectively [50]. Hence, in Australia where RSV prevalence is high, especially during the winter months, the number of false positives is assumed to be low. This is in contrast to earlier studies, which have relied on ICD-10-AM diagnostic codes for RSV-associated illnesses. Previous studies have found that RSV was identified across multiple ICD-10-AM diagnostic codes and that using only ICD diagnostic codes is likely to be insufficient to accurately estimate disease burden, instead grossly under-estimating burden [51].

### Limitations

This review is not without its limitations. These included; (1) studies included mixed cohorts of both healthy and children at higher risk of disease (E.g. Included both ex-premature and term infants, and infants with and without comorbidities). Only one study stratified analyses by a priori risk. Lack of risk stratification may have resulted in confounding of the observed effect, due to greater representation of higher risk infants; (2) we were unable to delineate between RSV mono- and co-infections as a number of studies did not specify whether additional pathogens were detected in addition to RSV. Co-infection of RSV with other viruses or bacteria may have confounded the results, leading to a more severe clinical illness. For example, RSV co-infection with pneumococcal disease or human metapneumovirus has been associated with increased clinical severity and increased risk of ICU admission [52, 53]; (3) a meta-analysis was not undertaken due to methodological heterogeneity. The included articles demonstrated a mix of different age-group comparisons and measured outcomes, which were not amenable to standard meta-analysis; (4) none of the identified studies examined whether there was a statistically significant difference in the measured outcome between infants aged less than 6 months, compared to other age groups. Although there were observable marked differences in age-related proportions and rates, we cannot draw conclusions about statistical significance; (5) the contribution of other confounding factors including season of birth, household crowding, number of siblings, passive exposure to cigarette smoking were not able to be examined due to lack of data in the literature; (6) measures of disease burden are limited to diagnosed RSV, which primarily occurs within clinical inpatient settings, thus failing to account for the large majority of RSV which would be occurring undiagnosed in the community. Thus, disease burden is likely to be significantly higher than what is feasibly measurable and estimates are likely grossly lower than true burden; (7) findings may not hold true in low or middle income countries; (8) the data is weighted towards WA and NSW, with the remaining states and territories under-represented. Higher proportions of Aboriginal and Torres Strait Islander people relative to total population in NT, QLD and WA [54] has likely contributed to under-reporting of RSV infections in this population; (9) there is likely overlap of data reported in studies conducted in WA and Australia-wide; and (10) examining RSV counts or proportions of total infections may reflect a bias towards testing younger infants, due to their propensity to develop more severe disease. However, this limitation is addressed partly by examining RSV positivity and incidence rates in different age groups.

## Conclusion

This systematic review found that Australian infants aged 0–6 months are at increased risk of contracting RSV and are more likely to develop severe disease requiring hospitalisation and respiratory support, compared to children and adults of all ages. This is consistent with the global age-related trends. Furthermore, our findings verified that RSV incidence, hospitalisation and severity are greater in Aboriginal and Torres Strait Islander infants aged 0–6 months, compared to non-Indigenous infants. Lastly, age-related patterns persisted despite seasonal transmission variability and during the SARS-CoV-2 pandemic. These findings have important implications for rollout of active and passive immunisation programs, both at a national and global level. Maternal RSV vaccination during pregnancy as part of standard antenatal care is a promising strategy for widespread, easily accessible and effective protection against severe RSV disease in infancy. Additionally, routine administration of a single long-acting RSV antibody to infants prior to their first winter season could further contribute to substantially reducing disease burden in the first 6 months of life. These immunisation strategies would, in turn, significantly reduce the strain placed on the healthcare system via reduction in hospitalisations, ICU admissions, respiratory support requirements and emergency department presentations. The healthcare cost savings will likely offset the cost of a routine immunisation program; however, this requires a formal cost-benefit analysis to confirm.

### Electronic supplementary material

Below is the link to the electronic supplementary material.

Additional File 1: Pubmed Search Strategy

Additional File 2: Study Screening Questionnaire

Additional File 3: Standardised data extraction questionnaire for eligible studies included in review

Additional File 4: Critical Appraisal Skills Programme (CASP) questionnaire results

Additional File 5: PRISMA Checklist

## Data Availability

All data generated or analysed during this study are included in this published article [see Additional files 3 and 4].

## List of Abbreviations

ALRI: Acute lower respiratory tract infection
BiPAP: Bilevel positive airway pressure
BPD: Bronchopulmonary dysplasia
CASP: Critical Appraisal Skills Programme
CI: Confidence interval
CPAP: Continuous positive airway pressure
ED: Emergency department
HDU: High dependency unit
HFNP: High flow nasal prongs
ICU: Intensive care unit
IQR: Interquartile range
IRR: Incidence rate ratio
IV: Intravenous
LBW: Low birth weight
NG: Nasogastric
NICU: Neonatal intensive care unit
NSW: New South Wales
NT: Northern Territory
OR: Odds ratio
PCR: Polymerase chain reaction
PRISMA: Preferred Reporting Items for Systematic Reviews and Meta-Analyses
QLD: Queensland
RCT: Randomised controlled trial
RSV: Respiratory syncytial virus
RSV-mAb: Maternal antibodies against RSV
RSVpreF: RSV prefusion F protein-based vaccine
SA: South Australia
SARS-CoV-2: Severe Acute Respiratory Syndrome Coronavirus 2
SD: Standard deviation
SWiM: Synthesis without meta-analysis
US: United States
VIC: Victoria
WA: Western Australia
WHO: World Health Organisation
