# Supplementary material for: Respiratory syncytial virus disease morbidity in Australian infants aged 0 to 6 months: a systematic review with narrative synthesis
Source: BMC Public Health. 2023 Dec 21;23:2560. doi: 10.1186/s12889-023-17474-x (PMC10740277; doi:10.1186/s12889-023-17474-x)
Supplement: Supplementary file 1 — Additional File 1: Pubmed Search Strategy [file 12889_2023_17474_MOESM1_ESM.docx]

ADDITIONAL FILE 1

**Pubmed Search Strategy**

(respiratory syncytial OR RSV)

AND

(morbidity OR mortality OR hospital* OR “intensive care” OR “critical care” OR hypoxia OR “oxygen inhalation therapy” OR oxygen OR “artificial respiration” OR emergency OR “community medicine” OR "cost of illness" OR “risk factors” OR Palivizumab)

AND

(Australia* [Title/Abstract])

**Ovid Search Strategy (Medline / Embase / Global Health)**

1. exp respiratory syncytial viruses/ or exp respiratory syncytial virus, human/

2. Respiratory Syncytial Virus Infections/

3. respiratory syncytial vir*.mp.

4. RSV.mp.

5. 1 or 2 or 3 or 4

6. exp morbidity/ or exp mortality/

7. morbidity.mp.

8. mortality.mp.

9. exp Hospitalization/

10. hospitali#ation.mp.

11. exp Intensive Care Units/ or exp Critical Care/

12. Hypoxia/ or Oxygen Inhalation Therapy/ or Oxygen/

13. exp Respiration, Artificial/

14. Emergency Service, Hospital/

15. Community Medicine/

16. exp "Cost of Illness"/

17. Risk Factors/

18. Palivizumab/

19. 6 or 7 or 8 or 9 or 10 or 11 or 12 or 13 or 14 or 15 or 16 or 17 or 18

20. Australia*.mp.

21. exp Australia/

22. 20 or 21

23. 5 and 19 and 22

24. limit 23 to (english language and yr="2000 - 2023")

25. remove duplicates from 24
