# Supplementary material for: Respiratory syncytial virus disease morbidity in Australian infants aged 0 to 6 months: a systematic review with narrative synthesis
Source: BMC Public Health. 2023 Dec 21;23:2560. doi: 10.1186/s12889-023-17474-x (PMC10740277; doi:10.1186/s12889-023-17474-x)
Supplement: Supplementary file 2 — Additional File 2: Study Screening Questionnaire [file 12889_2023_17474_MOESM2_ESM.docx]

ADDITIONAL FILE 2

**Study Screening Questionnaire**

| Study ID |  |
| --- | --- |
| Publication Year |  |
| Authors |  |
| Title |  |
| Relevancy based on title / abstract | Relevant / Irrelevant |
| Type of study |  |
| Age group of population |  |
| Location of study |  |
| Years of data collection |  |
| Laboratory confirmation of RSV |  |
| RSV-A, RSV-B, RSV unspecified |  |
| Study design |  |
| Included / Excluded | Included / Excluded |
| Reason excluded |  |
| Discrepancy between AS and JC | Yes / No |
| Resolution of discrepancy | Included / Excluded |
