# Supplementary material for: Respiratory syncytial virus disease morbidity in Australian infants aged 0 to 6 months: a systematic review with narrative synthesis
Source: BMC Public Health. 2023 Dec 21;23:2560. doi: 10.1186/s12889-023-17474-x (PMC10740277; doi:10.1186/s12889-023-17474-x)
Supplement: Supplementary file 4 — Additional File 4: Critical Appraisal Skills Programme (CASP) questionnaire results [file 12889_2023_17474_MOESM4_ESM.docx]

ADDITIONAL FILE 4

**Critical Appraisal Skills Programme (CASP) questionnaire results**

| **Study** | **1** | **2** | **3** | **4** | **5a** | **5b** | **6a** | **6b** | **7** | **8** | **9** | **10** | **11** | **12** |
| --- | --- | --- | --- | --- | --- | --- | --- | --- | --- | --- | --- | --- | --- | --- |
| Moore et al. (2020) (23) | 1 | 1 | 1 | 1 | 0 | 0.5 | 1 | 1 | NA | 1 | 1 | 1 | 1 | 1 |
| Homaira et al. (2019) (29) | 1 | 1 | 1 | 1 | 1 | 1 | 1 | 1 | NA | 1 | 1 | 1 | 1 | 1 |
| Gebremedhin et al. (2022) (22) | 1 | 1 | 1 | 1 | 1 | 1 | 0.5 | 1 | NA | 1 | 1 | 1 | 1 | 1 |
| Moore et al. (2019) (25) | 1 | 1 | 1 | 1 | 1 | 1 | 0.5 | 1 | NA | 0.5 | 1 | 1 | 1 | 1 |
| Anderson et al. (2021) (37) | 1 | 1 | 1 | 1 | 1 | 1 | 0.5 | 0 | NA | 1 | 1 | 1 | 1 | 1 |
| Homaira et al. (2016) (31) | 1 | 1 | 1 | 1 | 0.5 | 0.5 | 0.5 | 1 | NA | 1 | 1 | 1 | 1 | 1 |
| Lim et al. (2017) (32) | 1 | 1 | 1 | 1 | 1 | 1 | 0.5 | 1 | NA | 1 | 1 | 1 | 1 | 1 |
| Dede et al. (2010) (34) | 1 | 1 | 1 | 1 | 0 | 0 | 0.5 | 1 | NA | 0.5 | 1 | 1 | 1 | 1 |
| Saravanos et al. (2019) (3) | 1 | 1 | 1 | 1 | 1 | 1 | 0.5 | 1 | NA | 1 | 1 | 1 | 1 | 1 |
| Saravanos et al. (2022) (24) | 1 | 1 | 1 | 1 | 1 | 1 | 0.5 | 1 | NA | 1 | 1 | 1 | 1 | 1 |
| Fagan et al. (2017) (26) | 1 | 1 | 1 | 1 | 0 | 0 | 0.5 | 0 | NA | 0 | 1 | 1 | 1 | 1 |
| Butler et al. (2019) (30) | 1 | 1 | 1 | 1 | 1 | 1 | 0.5 | 0 | NA | 1 | 1 | 1 | 1 | 1 |
| Pham et al. (2020) (36) | 1 | 1 | 1 | 1 | 1 | 1 | 0.5 | 1 | NA | 1 | 1 | 1 | 1 | 1 |
| Fathima et al. (2018) (33) | 1 | 1 | 1 | 1 | 0.5 | 0 | 0.5 | 1 | NA | 1 | 1 | 1 | 1 | 1 |
| Nguyen et al. (2023) (35) | 1 | 1 | 1 | 1 | 0.5 | 0 | 0.5 | 1 | NA | 0.5 | 1 | 1 | 1 | 1 |
| Moore et al. (2012) (27) | 1 | 1 | 1 | 1 | 1 | 1 | 1 | 1 | NA | 0.5 | 1 | 1 | 1 | 1 |
| Chappell et al. (2013) (28) | 1 | 1 | 1 | 1 | 1 | 1 | 1 | 0 | NA | 0.5 | 1 | 0 | 1 | 1 |

Items assessed were; (1) Did the results address a clearly focused issue? (2) Was the cohort recruited in an acceptable way? (3) Was the exposure accurately measured to minimise bias? (4) . Was the outcome accurately measured to minimise bias? (5a) Have the authors identified all important confounding factors? (5b) Have they taken account of the confounding factors in the design and/or analysis? (6a) Was the follow up of subjects complete enough? (6b) Was the follow up of subjects long enough? (7) What are the results of this study? (8) Are the results precise? (9) Do you believe the results? (10) Can the results be applied to the local population? (11) Do the results of this study fit with other available evidence? (12) What are the implications of this study for practice?.
